# Scheduled Inside Plastic Stent Exchange Prevents Cholangitis and Reduces Unplanned Hospitalization in Patients With Unresectable Malignant Hilar Biliary Obstruction

**DOI:** 10.1002/deo2.70242

**Published:** 2025-11-10

**Authors:** Joji Muramatsu, Kazuma Ishikawa, Norito Suzuki, Tomohiro Kubo, Makoto Yoshida, Ginji Omori, Ryo Ito, Shogo Miura, Kohichi Takada

**Affiliations:** ^1^ Division of Medical Oncology, Department of Internal Medicine Sapporo Medical University School of Medicine Hokkaido Japan; ^2^ Department of Gastroenterology Oji General Hospital Hokkaido Japan; ^3^ Department of Gastroenterology Otaru Ekisaikai Hospital Hokkaido Japan; ^4^ Department of Gastroenterology Rumoi City Hospital Hokkaido Japan

**Keywords:** biliary stent, biliary tract cancer, cholangitis, ERCP, scheduled stent exchange

## Abstract

**Objectives:**

To prevent cholangitis in patients with unresectable malignant hilar biliary obstruction (MHBO), we recently implemented scheduled inside plastic stent (IS) exchange every 2–4 months. This study aimed to evaluate whether this strategy prevents cholangitis onset and reduces unplanned hospitalizations without increasing adverse events.

**Methods:**

This retrospective single‐center study included patients with unresectable MHBO who underwent IS placement between 2011 and 2023. Patients were divided into two groups: those who underwent scheduled IS exchange (scheduled group, *n* = 12) and those who received on‐demand IS exchange (on‐demand group, *n* = 29). We compared unplanned hospitalization duration and number, time from initial IS placement to cholangitis onset, 1‐year cholangitis‐free rate from initial IS placement, cholangitis severity, adverse events, medical costs, duration of antitumor therapy, and prognosis.

**Results:**

Median duration of unplanned hospitalizations was significantly shorter in the scheduled group compared to the on‐demand group (0 days [range: 0–83] vs. 38.5 days [0–140], *p* = 0.03). The time from initial IS placement to the onset of cholangitis was also significantly longer in the scheduled group (median 203 days [range: 54–463] vs. 89 days [4–1081], *p* = 0.02). The 1‐year cholangitis‐free rate was significantly higher in the scheduled group (50% vs. 10%, *p* = 0.01). No significant differences were found in the number of unplanned hospitalizations, cholangitis severity, adverse events, medical costs, duration of antitumor therapy, or prognosis.

**Conclusions:**

Scheduled IS exchange delays the onset of cholangitis and reduces the duration of unplanned hospitalizations without increasing adverse events.

## 1 Introduction

Endoscopic biliary drainage (EBD) is commonly performed in patients with unresectable malignant hilar biliary obstruction (MHBO). Two types of stents are used in EBD: plastic stents (PS) and metallic stents (MS). While MS generally provides a longer patency period than PS, uncovered MS—frequently used in the hilar region—cannot be removed, making reintervention challenging in cases of occlusion. Historically, the prognosis for unresectable MHBO has not significantly exceeded the patency duration of MS (4–8 months) [1, 2, 3], and thus, few patients required reintervention. With advances in antitumor therapies, however, patient survival is improving, leading to an increasing number of reinterventions following MS placement. By contrast, PS can be readily removed if occlusion occurs. In particular, inside PS (IS), which are placed above the papilla, offer longer patency than PS placed across the papilla [4]. IS placement, therefore, is our preferred approach for biliary drainage in patients with MHBO. The time to recurrent biliary obstruction (RBO) [5] for IS, however, is typically around 3–4 months [6, 7, 8, 9]. IS occlusion or cholangitis can result in unplanned hospitalizations and may interrupt or delay antitumor treatment, negatively impacting both patients and healthcare providers.

We previously performed IS exchange on demand in cases of occlusion (data not shown). More recently, however, we have adopted a scheduled IS exchange protocol every 2–4 months to proactively prevent cholangitis. To date, however, no studies have demonstrated that scheduled IS exchange reduces the incidence of cholangitis. In this study, we aimed to evaluate whether scheduled IS exchange in patients with unresectable MHBO can prevent cholangitis and reduce unplanned hospitalizations without increasing the incidence of adverse events.

## 2 Methods

### 2.1 Study Approval

This study was approved by the Ethics Committee of Sapporo Medical University Hospital (IRB number: 352‐127) and conducted in accordance with the Declaration of Helsinki. Written informed consent was obtained from all patients prior to undergoing endoscopic retrograde cholangiopancreatography (ERCP). Patients were given the opportunity to opt out through a notice posted on the hospital's website.

### 2.2 Patients

This was a single‐center, retrospective study of patients with unresectable MHBO, histologically confirmed as adenocarcinoma, who underwent IS placement at Sapporo Medical University Hospital between January 2011 and June 2023. Patients with a history of gastrectomy other than Billroth I reconstruction, those who underwent short‐term IS placement as a bridge to MS placement, and those who received mixed strategies of on‐demand and scheduled IS exchanges during the clinical course were excluded. Information on the stent exchange strategy was obtained from electronic medical records.

### 2.3 ERCP Procedures

Endoscopic sphincterotomy, biopsy of the stricture site, and biliary drainage using endoscopic nasobiliary drainage (ENBD) were performed during the initial ERCP. After resolution of jaundice, control of cholangitis, and confirmation of a pathological diagnosis, a 7Fr biliary PS (9 or 12 cm, Through & Pass Inside Stent; Gadelius Medical, Tokyo, Japan) was placed above the papilla in a subsequent session. The number of IS placed was determined by the physician to achieve drainage of >50% of the liver volume. In case of stent dysfunction after IS placement, the stents were replaced with either new IS or ENBD at the physician's discretion. ENBD was subsequently exchanged for a new IS after resolution of cholangitis. The clinical pathway for scheduled IS exchange is a 4‐day hospitalization: ERCP on day 2, oral intake resumed on day 3 if no complications, and discharge on day 4.

### 2.4 Outcome Measurements and Definitions

The primary outcome was the total duration and number of unplanned hospitalizations due to cholangitis over a patient's lifetime. Secondary outcomes included: time from initial IS placement to the onset of cholangitis, the 1‐year cholangitis‐free rate from initial IS placement, severity of the initial cholangitis, clinical factors associated with the onset of cholangitis, total number of ERCP, and duration of hospitalization associated with all biliary interventions over a patient's lifetime, type of biliary interventions, adverse events, medical costs, duration of antitumor therapy, and overall survival (OS).

The scheduled IS exchange group was defined as patients in whom ERCP was scheduled before the onset of cholangitis, while the on‐demand IS exchange group was defined as those patients who did not undergo ERCP until cholangitis developed. Technical success and clinical success were determined according to Tokyo Criteria 2024 [5]. Failure of the scheduled IS exchange was defined as the occurrence of cholangitis prior to the scheduled procedure.

**FIGURE 1:**
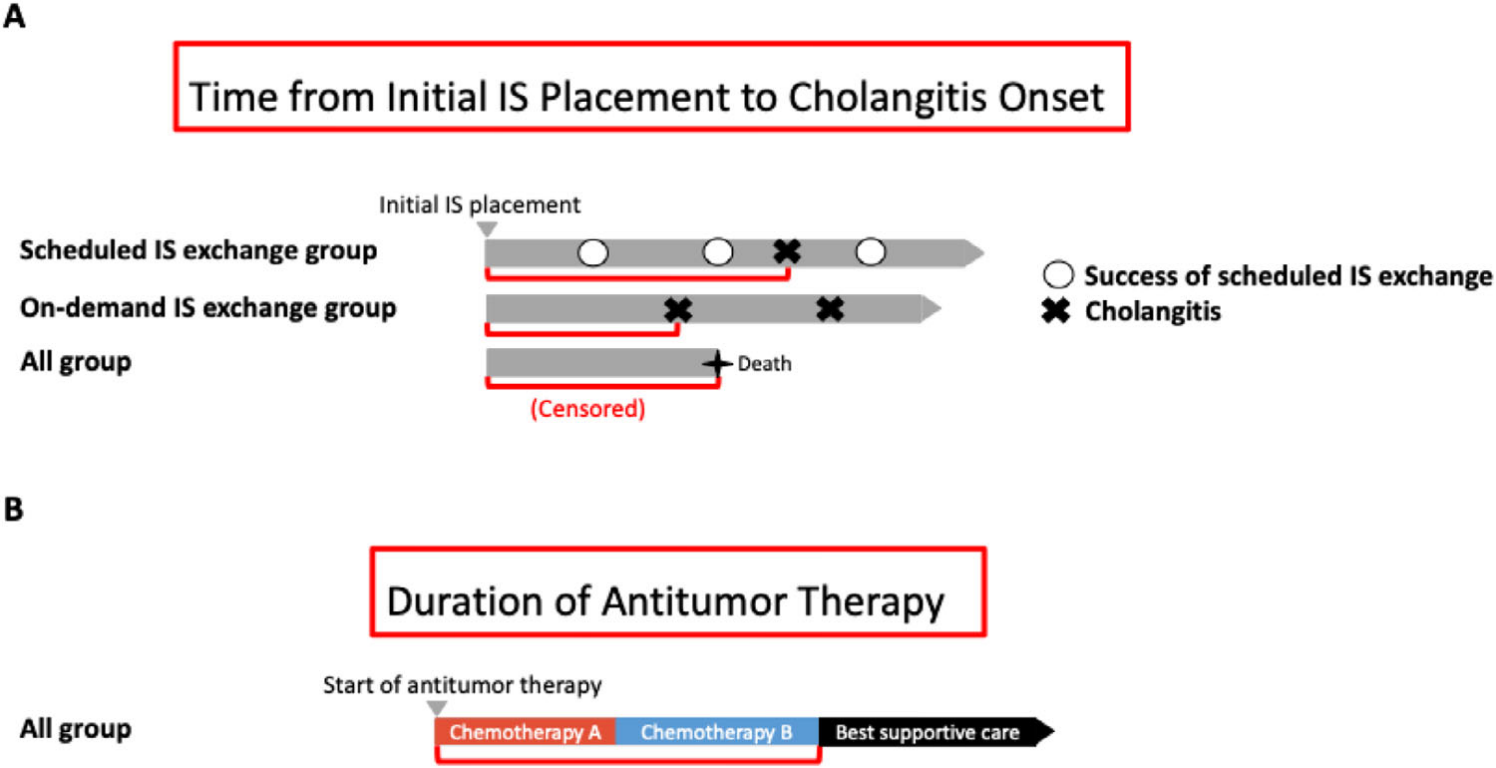
Definitions used in this study. (A) Time from initial IS placement to cholangitis onset (red lines) in both the scheduled IS exchange and on‐demand IS exchange groups. Grey arrowheads represent the duration of patient follow‐up from initial IS placement to death or censoring. (B) Duration of antitumor therapy (red lines) in all patients. IS, inside plastic stent.

Cholangitis diagnosis and severity were evaluated using the Tokyo Guidelines 2018 [10]. The time from initial IS placement to cholangitis was defined as the interval from the first IS placement to the onset of cholangitis. Scheduled IS exchange was not considered a censored event, and the observation period continued accordingly (Figure 1A). Patients lost to follow‐up due to transfer were censored at the last confirmed date of IS patency, and patients who died before developing cholangitis were censored at the date of death. The number of endoscopic biliary interventions was assessed according to Tokyo Criteria 2024. If ENBD was placed during cholangitis and subsequently converted to IS, these two procedures were counted as one biliary intervention.

Clinical factors associated with the onset of cholangitis were analyzed, including: age, sex, Bismuth classification, lymph node metastasis, distant metastasis, CA19‐9 levels at diagnosis, details of the antitumor therapy used, best overall computed tomography response, history of cholangitis before initial ERCP, ENBD placement prior to IS placement, bile sludge observed on cholangiography prior to initial cholangitis, bilateral drainage at initial IS placement, number of IS at initial placement, IS exchange strategy (on‐demand or scheduled), and bile culture results at the time of the initial cholangitis.

Adverse events and their severity were evaluated using Lexicon criteria [11] and Tokyo Criteria 2024. Non‐occlusion cholangitis was defined as cholangitis that could be managed conservatively without biliary intervention. Non‐occlusion cholangitis associated with endoscopic procedures was not considered the onset of cholangitis. Medical costs were calculated based on insurance reimbursement points relating to total hospitalization expenses associated with all biliary interventions.

The duration of antitumor therapy was defined as the period from initiation of therapy to the decision to convert to best supportive care (Figure 1B). For patients lost to follow‐up during this period, the last treatment date was used as the censoring point. OS was defined as the time from pathological diagnosis to death, with patients lost to follow‐up censored at the last confirmed survival date.

### 2.5 Statistical Analysis

Fisher's exact test was used to compare categorical variables, and either Student's *t*‐test or the Mann–Whitney *U* test was used for continuous variables. Time from initial IS placement to cholangitis, duration of antitumor therapy, and OS were estimated using the Kaplan–Meier method and compared with the log‐rank test. Univariate analysis of clinical factors associated with the onset of cholangitis was also performed using the Kaplan–Meier method and log‐rank test, and hazard ratios were estimated using the Cox regression model. Multivariate analysis was conducted using the Cox regression analysis as well. All statistical analyses were performed using EZR software (version 1.55) [12], with a *p*‐value of <0.05 considered statistically significant.

## 3 Results

### 3.1 Patient Characteristics

We enrolled 41 patients in this study: 12 in the scheduled IS exchange group (scheduled group) and 29 in the on‐demand IS exchange group (on‐demand group; Table 1). We saw no significant differences in age or sex distribution between groups, and most patients were diagnosed with hilar cholangiocarcinoma. We also found that the proportions of patients with Bismuth classifications, lymph node, and distant metastases were similar between the groups.

**TABLE 1:** Patient characteristics.

	Scheduled group (*n* = 12)	On‐demand group (*n* = 29)	*p*‐Value
Median age in years (range)	73 (58–78)	75 (55–86)	0.36
Sex, *n* (%)			0.73
Male	7 (58%)	19 (66%)	
Female	5 (42%)	10 (34%)	
Primary tumor site, *n* (%)			0.65
Perihilar bile duct	9 (75%)	17 (59%)	
Gallbladder	3 (25%)	7 (24%)	
Intrahepatic bile duct	0	4 (14%)	
Extensive bile duct	0	1 (3%)	
Bismuth classification, *n* (%)			0.07
I–II	2 (17%)	15 (52%)	
III–IV	10 (83%)	14 (48%)	
Lymph node metastasis, *n* (%)			0.73
Positive	5 (42%)	10 (34%)	
Negative	7 (58%)	19 (66%)	
Distant metastasis, *n* (%)			1.00
Positive	2 (17%)	6 (21%)	
Negative	10 (83%)	23 (79%)	
Antitumor therapy, *n* (%)			0.65
Yes	11 (92%)	24 (83%)	
No	1 (8%)	5 (17%)	
First‐line therapy, *n* (%)			**0.0014**
GCD, GCS	8 (73%)	3 (12%)	
GC, GS	2 (18%)	17 (71%)	
CRT	1 (9%)	4 (17%)	

Abbreviations: CRT, chemoradiotherapy; GC, Gemcitabine + Cisplatin; GCD, Gemcitabine + Cisplatin + Durvalumab; GCS, Gemcitabine + Cisplatin + S‐1GS, Gemcitabine + S‐1.

Antitumor therapy was initiated in 80%–90% of patients in both groups (Table 1). As we expected from changes in clinical practice over time, gemcitabine + cisplatin + durvalumab (GCD) therapy or gemcitabine + cisplatin + S‐1 (GCS) therapy was predominantly used in the scheduled group.

### 3.2 Impact of Scheduled IS Exchange on Cholangitis Prevention and Unplanned Hospitalizations

**FIGURE 2:**
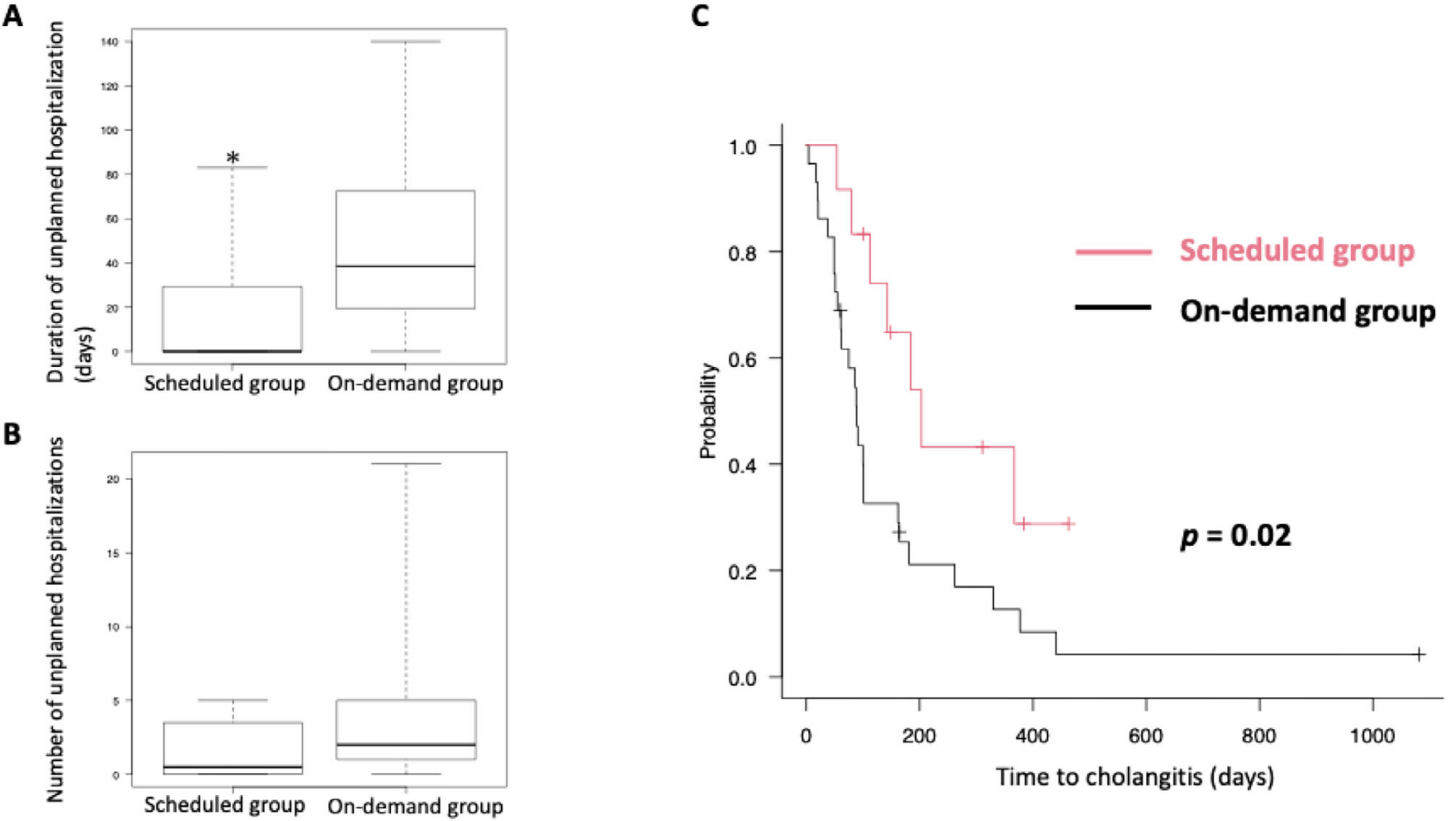
Effect of scheduled IS exchange on preventing cholangitis compared to on‐demand exchange. (A, B) Mann–Whitney *U* test results for the total duration (A) and number (B) of unplanned hospitalizations due to cholangitis over a patient's lifetime. In the box‐and‐whisker plot, the bottom of the box represents the first quartile of the data, and the top of the box represents the third quartile. The line inside the box indicates the median. The “whiskers” extending above and below the box represent the full range of the data. (C) Kaplan–Meier curves showing time to cholangitis after initial IS placement in the scheduled and on‐demand groups. *p < 0.05. IS, inside plastic stent.

The median duration of unplanned hospitalizations due to cholangitis was significantly shorter in the scheduled group compared to the on‐demand group (0 days [range: 0–83] vs. 38.5 days [0–140], *p* = 0.03, Figure 2A). Although there was a trend toward fewer such hospitalizations in the scheduled group, the median number was not significantly different (0.5 [range: 0–5] vs. 2 [0–21], *p* = 0.05, Figure 2B). Time from initial IS placement to cholangitis onset was longer in the scheduled group (median 203 days [range: 54–463] vs. 89 days [4–1081], *p* = 0.02, Figure 2C). The 1‐year cholangitis‐free rate was higher in the scheduled group (50% vs. 10%, *p* = 0.01, Table 2). The proportion of moderate/severe cholangitis cases was similar (33% vs. 43%, Table 2).

**TABLE 2:** Incidence and severity of cholangitis within 1 year from initial inside plastic stent (IS) placement.

	Scheduled group (*n* = 12)	On‐demand group (*n* = 29)	*p*‐Value
Onset of the initial cholangitis, *n* (%)			0.01
Yes	6 (50%)	26 (90%)	
No	6 (50%)	3 (10%)	
Severity of first cholangitis, *n* (%)			1.00
Mild	4 (67%)	15 (57%)	
Moderate‐Severe	2 (33%)	11 (43%)	

Abbreviation: IS, inside plastic stent.

In univariate analysis, the following factors were significantly associated with the time to cholangitis: absence of GCD or GCS therapy (hazard ratio [HR] = 3.2), best overall response less than partial response (PR) (HR = 5.6), presence of cholangitis before initial ERCP (HR = 3.1), use of three or more stents at initial IS placement (HR = 2.9), on‐demand IS exchange (HR = 2.5), and detection of *Candida* spp. in bile culture (HR = 3.4, Table 3). GCS or GCD therapy was excluded from multivariate analysis due to a moderate correlation with the best overall response using Spearman correlation analysis [13] (r = 0.51, *p* = 0.001). Cox regression using a forward‐backward stepwise selection method based on Akaike's Information Criterion identified three factors for inclusion: best overall response less than PR, use of three or more stents, and on‐demand IS exchange. All three factors were statistically significant, with hazard ratios of 14.3, 3.9, and 3.2, respectively (Table 3).

**TABLE 3:** Clinical factors associated with the onset of cholangitis.

	Univariate HR (95% CI)	*p*‐Value	Multivariate HR (95% CI)	*p*‐Value
Age > 80	1.47 (0.60–3.56)	0.39		
Male	1.40 (0.69–2.85)	0.34		
Bismuth classification III–IV	0.92 (0.47–1.81)	0.81		
Lymph node metastasis positive	0.79 (0.39–1.60)	0.52		
Distant metastasis positive	1.01 (0.41–2.45)	0.97		
CA19‐9 elevated at diagnosis	1.08 (0.52–2.21)	0.83		
GCD or GCS therapy not administered	3.20 (1.21–8.43)	**0.01**		
Best overall response SD or PD	5.67 (1.70–18.8)	**< 0.01**	14.3 (1.75–116)	**0.01**
Cholangitis before initial ERCP	3.18 (1.30–7.74)	**0.01**		
ENBD used before IS placement	1.62 (0.74–3.55)	0.22		
Bile sludge observed in cholangiography prior to the initial cholangitis	0.85 (0.20–3.66)	0.83		
Bilateral drainage at initial IS placement	1.08 (0.53–2.19)	0.83		
Three or more stents at initial IS placement	2.98 (1.17–7.58)	**0.02**	3.90 (1.23–12.3)	**0.02**
On‐demand IS exchange	2.54 (1.10–5.90)	**0.02**	3.20 (1.13–9.12)	**0.02**
Bacteria detected in bile culture at the initial cholangitis				
* Enterococcus* spp.	0.50 (0.22–1.13)	0.09		
* Streptococcus* spp.	0.76 (0.28–2.05)	0.59		
* Citrobacter* spp.	2.30 (0.77–6.87)	0.13		
* Clostridium* spp.	1.48 (0.50–4.33)	0.46		
* Enterobacter* spp.	1.79 (0.71–4.50)	0.21		
* Escherichia coli*	2.67 (0.34–20.9)	0.34		
* Klebsiella* spp.	0.81 (0.38–1.72)	0.59		
* Pseudomonas* spp.	0.40 (0.05–2.98)	0.37		
* Candida* spp.	3.46 (1.12–10.72)	**0.03**		

Abbreviations: CI, confidence interval; ERCP, endoscopic retrograde cholangiopancreatography; ENBD, endoscopic nasobiliary drainage; GCD, Gemcitabine + Cisplatin + Durvalumab; GCS, Gemcitabine + Cisplatin + S‐1; HR, hazard ratio; IS, inside plastic stent; PD, progressive disease; SD, stable disease; spp., species.

### 3.3 Endoscopic Interventions and Safety of Scheduled IS Exchange

The median observation period was 421 days in the scheduled group and 508 days in the on‐demand group, with no significant difference found (Table 4). The total number of ERCP procedures associated with all biliary interventions over a patient's lifetime was 6.5 in the scheduled group and 4 in the on‐demand group, without a statistically significant difference. Likewise, the total duration of hospitalization related to all biliary interventions was comparable between the two groups. In addition, the total number of bilateral drainages and the number of stents placed at initial IS placement were similar between the two groups.

**TABLE 4:** Details of endoscopic biliary interventions and associated adverse events.

	Scheduled group (*n* = 12)	On‐demand group (*n* = 29)	*p*‐Value
Median follow‐up period in days (range)	421 (123–646)	508 (73–1731)	0.26
Median number of ERCP associated with all biliary interventions (range)	6.5 (2–14)	4 (0–34)	0.17
Median number of scheduled exchanges (range)	3 (2–5)	—	
Median duration of hospitalization associated with all biliary interventions in days (range)	22.5 (8–87)	38.5 (0–140)	0.34
Bilateral drainage at initial IS placement, *n* (%)	9 (75%)	18 (62%)	0.49
Median number of stents at initial IS placement (range)	2 (1–3)	2 (1–3)	0.96
Total number of percutaneous interventions, *n* (%)	0	4 (13%)	
Severe stricture (overgrowth or new obstruction of the ducts)		3	
Insufficient endoscopic biliary drainage		1	
Total number of MS placements, *n* (%)	1 (8%)	5 (17%)	0.65
Median time to convert to MS from initial IS placement in days (range)	279 (279)	359 (49–568)	1.0
Total number of moderate or severe ERCP‐related adverse events			0.56
All, *n* (%)	2 (16%)	2 (6%)	
Non‐occlusion cholangitis	2	1	
Cholecystitis	0	1	

Abbreviations: ENBD, endoscopic nasobiliary drainage; ERCP, endoscopic retrograde cholangiopancreatography; IS, inside plastic stent; MS, metallic stent.

We considered the possibility that ENBD placement might prolong hospitalization. Therefore, we examined the number of cases where ENBD was performed during unplanned hospitalizations and the ratio of ENBD placements to cholangitis episodes (total ENBD/total cholangitis); however, no significant difference was observed between the two groups (Table S1).

Percutaneous interventions were not required in patients of the scheduled group, but were required in four cases of the on‐demand group. The reasons for performing percutaneous transhepatic biliary drainage (PTBD) were as follows: in three cases, endoscopic biliary drainage could not be performed due to severe stricture (overgrowth or new obstruction of the ducts); and, in one case, endoscopic biliary drainage was performed in the intended bile duct, but clinical success was not achieved. MS were placed in one case in the scheduled group and in five cases in the on‐demand group. The median time to MS conversion from initial IS placement was 279 days in the scheduled group and 359 days in the on‐demand group. All percutaneous interventions and MS placements were conducted after the first cholangitis episode for all patients.

There was no significant difference in the incidence of moderate or severe ERCP‐related adverse events (*p* = 0.56, Table 4). In the scheduled group, two cases of non‐occlusion cholangitis occurred following the procedure. In all cases, non‐occlusion cholangitis developed on the day after the ERCP procedure and was resolved with antibiotic therapy.

**FIGURE 3:**
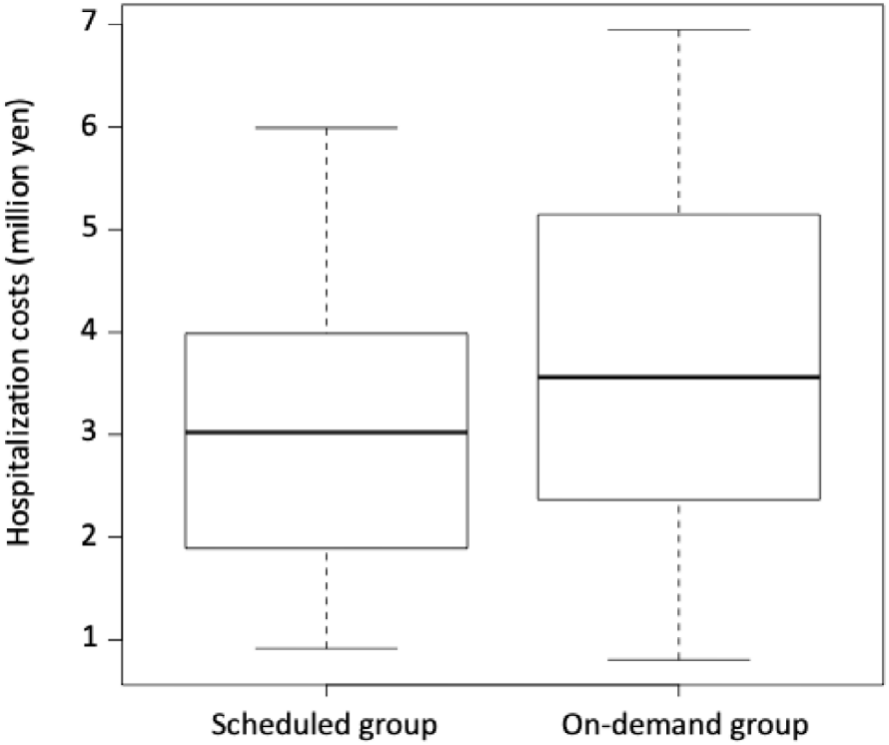
Total hospitalization expenses related to all biliary interventions over a patient's lifetime. In the box‐and‐whisker plot, the bottom of the box represents the first quartile of the data, and the top of the box represents the third quartile. The line inside the box indicates the median. The “whiskers” extending above and below the box represent the full range of the data.

The median medical costs were similar between groups: 3.0 million yen (range: 0.9–5.9) for patients in the scheduled group and 3.5 million yen (0.8–6.9) for those in the on‐demand group (Figure 3).

### 3.4 Antitumor Therapy Duration and Overall Survival

**FIGURE 4:**
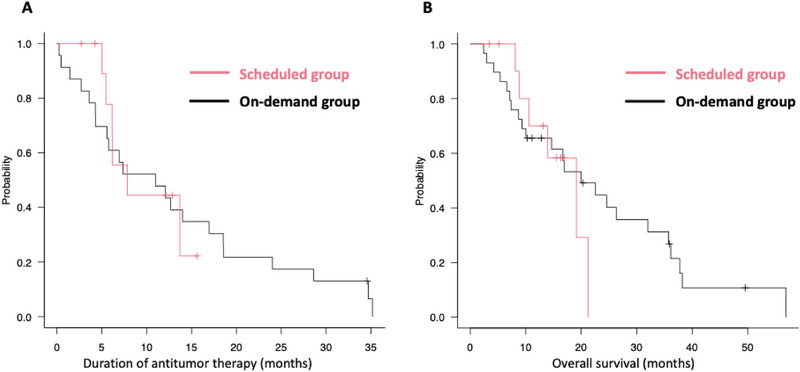
Duration of antitumor therapy and overall survival in scheduled and on‐demand groups. (A) Kaplan–Meier curves for the duration of antitumor therapy. (B) Kaplan–Meier curves for overall survival. IS, inside plastic stent.

No significant difference in antitumor therapy duration was observed: median 7.8 months (range: 2.7–15.8) in the scheduled group and 10.9 months (0.2–42.1) in the on‐demand group (Figure 4A). Overall survival was also similar: 19.1 months (3.4–21.5) vs. 19.9 months (2.4–57.7), respectively (Figure 4B). Six to seven patients in each group remained on treatment at the time of analysis.

### 3.5 Outcomes and Treatment Course in the Scheduled Group

**FIGURE 5:**
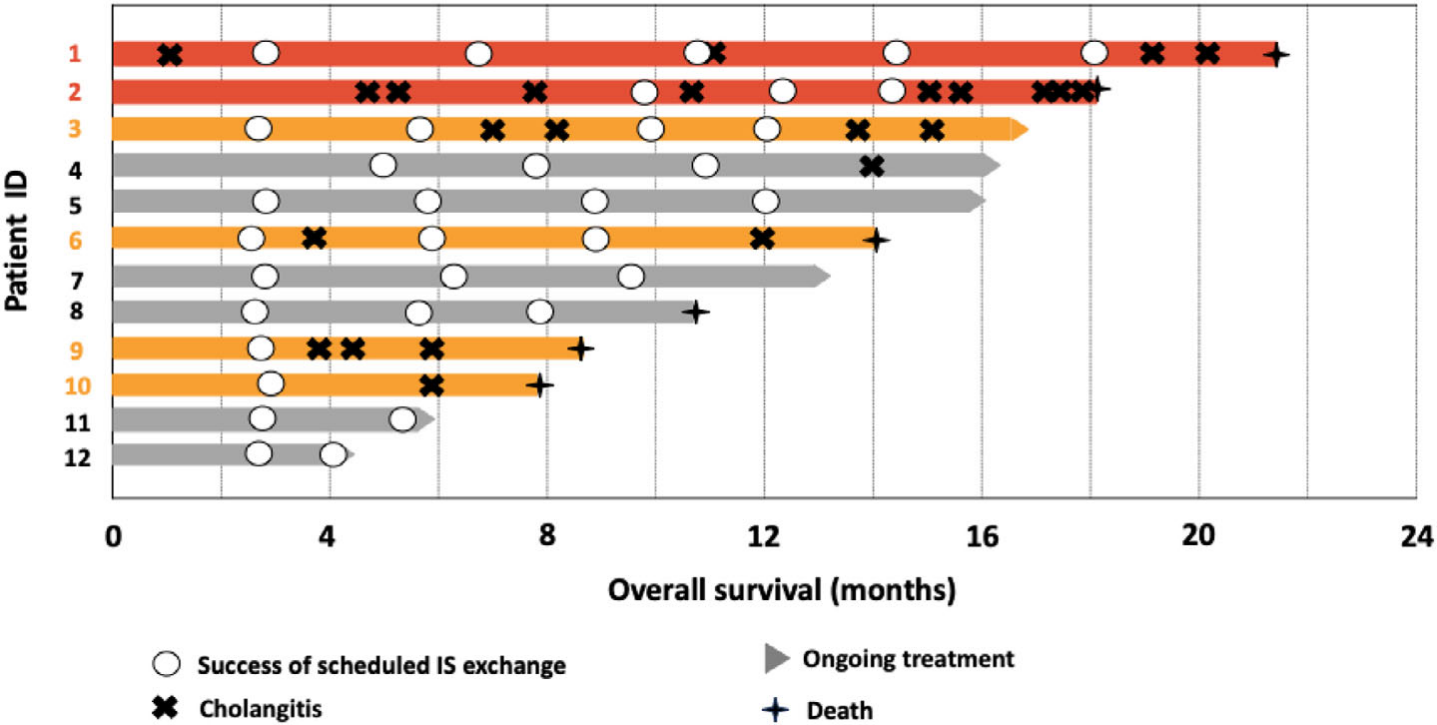
Swimmer plot of individual patient outcomes in the scheduled IS exchange group. Red lines: Cases that developed cholangitis between the initial IS placement and the first scheduled IS exchange. Orange lines: Cases that developed cholangitis within one year after initial IS placement. IS, inside plastic stent.

Of 12 patients in the scheduled group, the first scheduled IS exchange was successful in 10 of 12 patients (83%, Figure 5). Of the two cases of failed scheduled IS exchange (marked by red lines in Figure 5), cholangitis occurred at 54 days due to stent migration and at 143 days due to stent occlusion. Six patients (50%) experienced a failed scheduled IS exchange within 1 year of the initial IS placement (Table 2, Figure 5; red and orange lines).

In the scheduled group, among patients who successfully reached the scheduled IS exchange procedure, technical and clinical success rates were 100%. Although two cases of non‐occlusion cholangitis caused by scheduled IS exchange (Table 4), all improved with antibiotic therapy.

## 4 Discussion

Although previous studies have explored scheduled IS exchange for unresectable MHBO, its benefit remains unclear [7, 14, 15]. This study evaluated whether scheduled IS exchange reduces unplanned hospitalizations for cholangitis, maintains chemotherapy continuity, and affects costs. It prolonged the time to cholangitis, improved the 1‐year cholangitis‐free rate, and shortened unplanned hospital stays without increasing adverse events or ERCP frequency. Scheduled IS exchange may therefore be a safe and effective approach to prevent cholangitis.

To further explore strategies for improving the cholangitis‐free rate, we noted that the median time from initial IS placement to cholangitis onset in the on‐demand group was 89 days, suggesting that stent exchange within 3 months may be reasonable. Based on Figure 5, scheduling the first IS exchange at a 3‐month interval could enable successful exchange before cholangitis onset in over 90% of cases, which we consider a favorable outcome. Therefore, a 3‐month interval may serve as a useful benchmark for the first IS exchange.

During the first year after IS placement, 50% (6/12) of patients in the scheduled group experienced failure of a scheduled IS exchange. The interval between the preceding IS exchange and cholangitis onset ranged from 1 to 4 months, suggesting that second and subsequent IS exchanges may be inadequate if performed within 3 months. To determine the optimal interval, factors associated with early stent occlusion should be considered. Previous studies have identified advanced Bismuth classification, prior cholangitis, unresolved jaundice after stenting, and absence of chemotherapy as predictors of early occlusion; however, whether these also affect scheduled IS exchange failure remains unclear [16, 17, 18, 19]. In addition, time‐dependent factors such as stricture progression and sludge accumulation may increase failure risk, but could not be fully evaluated due to limited cases. Given the uncertainty of predictors and the potential shortening of optimal intervals over time, establishing a definitive benchmark for subsequent IS exchange intervals remains difficult.

From a quality of life (QOL) perspective, PTBD is important because it requires drained bile juice management and impairs QOL. In the scheduled group, no patients underwent PTBD. Scheduled IS exchange allowed assessment of stricture progression and additional drainage by increasing stent numbers before obstruction, which may explain the absence of PTBD, although the small sample precludes causal inference. Further studies with larger cohorts are needed to confirm whether scheduled exchange reduces PTBD and supports QOL maintenance.

No significant differences were observed between groups in chemotherapy duration or OS, consistent with previous reports showing no link between stent occlusion and prognosis in unresectable biliary tract cancers [20, 21]. Recent advances in ICI therapy have introduced new considerations, as antibiotic use may reduce ICI efficacy and worsen outcomes across cancers [22]. However, reports on biliary tract cancer remain conflicting [23]. Nevertheless, preventing cholangitis through scheduled IS exchange may reduce the need for antibiotics, potentially extending treatment duration and improving prognosis. Although prophylactic antibiotics were used during scheduled exchange in this study, future protocols will omit them to further support ICI effectiveness.

This study has several limitations. First, it was a single‐center, retrospective analysis with a small sample size and differences in baseline patient characteristics. A multicenter, collaborative study with larger cohorts and multivariate analysis is needed to validate these findings. Second, the observation period was longer than that in previous reports [24]. This may reflect selection bias, as the cohort included fewer patients with distant metastases and fewer receiving best supportive care. Recent improvements in prognosis with ICI may have contributed. Finally, we did not assess whether this approach improves QOL. To evaluate this, a prospective study including patient‐reported QOL measures is warranted.

## 5 Conclusion

Scheduled IS exchange can delay the onset of cholangitis and reduce unplanned hospitalizations in patients with unresectable MHBO compared to on‐demand IS exchange.

## Author Contributions

**Joji Muramatsu**: conceptualization (equal), data curation (equal), formal analysis (lead), investigation (equal), visualization (lead), and writing‐original draft preparation (lead). **Kazuma Ishikawa**: conceptualization (equal), data curation (equal), formal analysis (support), investigation (equal), visualization (support), and writing‐review & editing (lead). **Norito Suzuki**: investigation (equal), resources (equal), and validation (lead). **Tomohiro Kubo**: investigation (equal) and resources (equal). **Makoto Yoshida**: investigation (equal) and resources (equal). **Ginji Omori**: investigation (equal). **Ryo Ito**: investigation (equal). **Shogo Miura**: investigation (equal). **Kohichi Takada**: supervision (lead) and writing‐review & editing (support).

## Funding

No specific funding was received for the study.

## Ethics Statement

**Approval of the research protocol by an Institutional Reviewer Board (IRB)**: This study was approved by the Ethics Committee of Sapporo Medical University Hospital (IRB number: 352‐127).

## Consent

Written informed consent was obtained from all patients prior to undergoing ERCP. Patients were given the opportunity to opt out of participation through a notice posted on the hospital's website.

## Conflicts of Interest

Kazuma Ishikawa has received travel grants from THE ITO FUNDATION and THE JAPANESE FOUNDATION FOR RESEARCH AND PROMOTION OF ENDOSCOPY. Makoto Yoshida has received speaker honoraria from Daiichi Sankyo, MSD, and Novartis. Kohichi Takada has received speaker honoraria from Daiichi Sankyo, Chugai, Eisai, Janssen, Ono, MSD, Takeda, Sanofi, Eli Lilly, Zeria, and Sysmex.

## Clinical Trial Registration

Not applicable.

## Supporting information




**TABLE S1** Details of ENBD after initial IS placement. ENBD, endoscopic nasobiliary drainage; IS, inside plastic stent.
