# Supplementary material for: Scheduled Inside Plastic Stent Exchange Prevents Cholangitis and Reduces Unplanned Hospitalization in Patients With Unresectable Malignant Hilar Biliary Obstruction
Source: DEN Open. 2025 Nov 10;6(1):e70242. doi: 10.1002/deo2.70242 (PMC12599539; doi:10.1002/deo2.70242)
Supplement: Supplementary file 1 — TABLE S1 Details of ENBD after initial IS placement. ENBD, endoscopic nasobiliary drainage; IS, inside plastic stent. [file DEO2-6-e70242-s001.docx]

**Supplementary Table 1.**

|  | Scheduled group (n=12) | On-demand group (n=29) | *P*-value |
| --- | --- | --- | --- |
| Number of ENBD cases after initial IS placement, n (%) | 8 (67%) | 18 (62%) | 1.00 |
| Median ratio of ENBD placements to cholangitis | 38% | 42% | 0.85 |

Details of ENBD after initial IS placement

*ENBD, endoscopic nasobiliary drainage; IS, inside plastic stent.*
